# Detection of potentially curable recurrence after colorectal cancer surgery in a real-world population

**DOI:** 10.1371/journal.pone.0361933

**Published:** 2026-10-30

**Authors:** Ivonne J. H. Schoenaker, Richard M. Brohet, Evelyn J. Finnema, Dominique C. Damminga, Helle-Brit Fiebrich, Wouter H. de Vos tot Nederveen Cappel, Henderik L. van Westreenen

**Affiliations:** 1 Oncology Center Isala, Isala, Zwolle, the Netherlands; 2 Department of Health Science, Section of Nursing Research, University of Groningen, University Medical Center Groningen, Groningen, the Netherlands; 3 Department of Epidemiology & Statistics, Isala, Zwolle, the Netherlands; 4 Department of Gastroenterology and Hepatology, Isala, Zwolle, the Netherlands; 5 Department of Surgery, Isala, Zwolle, the Netherlands; Fujita Health University: Fujita Ika Daigaku, JAPAN

## Abstract

**Introduction:**

After curative surgery for colorectal cancer (CRC), patients undergo surveillance because of the risk of recurrence; however, only a subset develops recurrence amenable to further curative-intent treatment. The primary aim of follow‑up is to identify patients with potentially curable recurrence.

**Materials and methods:**

A retrospective analysis was performed of stage I–IV CRC patients who underwent curative-intent treatment and entered postoperative surveillance at Isala between 2014 and 2018. Follow‑up was conducted according to Dutch guideline‑based protocols for 60 months. Patients were categorized as having no recurrence, a potentially curable recurrence, or a non-curable recurrence. Prognostic factors at start of follow-up for potentially curable recurrence were assessed using competing‑risk Cox regression, with non-curable recurrence and death as competing events. For patients with recurrence, mode and time of detection were evaluated.

**Results:**

During a median follow-up of 60 months, recurrence occurred in 22.4% of 1118 patients. The cumulative incidence function (CIF) of a potentially curable recurrence was 8.0%. Independent variables associated with a potentially curable recurrence were age HR 0.97 (95% CI 0.96–0.99), minor postoperative complications HR 1.78 (95% CI 1.16–2.72), stage III CRC HR2.76 (95% CI 1.19–6.38) and stage IV CRC HR 11.02 (95% CI 4.44–27.37). Among patients with a recurrence, CEA ≥ 5 ng/mL was present in 63% of all recurrences, but less often in the potentially curable group (57% vs. 69%, p = 0.057). Median time to detection of a potentially curable recurrence was 16 months (IQR 20). The overall number needed to follow-up (NNFU) to detect one potentially curable recurrence was 9.8, with the lowest NNFU observed in stage IV CRC (2.3) and in rectal cancer (6.9).

**Conclusion:**

Standard follow-up enabled the detection of potentially curable recurrence in 8% of CRC patients. The substantial variation in NNFU across subgroups and the limited sensitivity of CEA underscore the need for more personalized, risk‑adapted surveillance strategies.

## Introduction

Colorectal cancer (CRC) is the third most common cancer worldwide, with an estimated 12,000 new cases diagnosed annually in the Netherlands [1,2]. The introduction of the national CRC screening program in 2014 has led to earlier detection and reduced incidence. Nevertheless, the absolute numbers of CRC cases are expected to rise due to an aging population.

The main goal of follow-up after curative resection of CRC is the early detection of asymptomatic recurrences that are eligible for curative treatment, with the intention to improve survival [3]. Recurrence occurs in 20–30% of patients with stage I–III CRC, most frequently within the first three years post-treatment and most commonly in the liver, lungs, or peritoneum [4,5]. Although several studies have evaluated different follow-up strategies, intensified surveillance has not demonstrated an improvement in overall survival at a population level, even though it leads to earlier detection and increases the number of patients receiving treatment for recurrence [6–9]. In the COLOFOL trial, 9,6% of patients developed liver metastases, of whom 52,3% underwent treatment with curative intent, resulting in a five‑year overall survival rate of 58% [10]. Similarly, reported five-year survival rates after pulmonary metastasectomy range from 21% to 61.4% [11].

International guidelines generally recommend standardized follow-up for stage I-III CRC, including regular Carcino-Embryonic Antigen (CEA) testing, imaging, and colonoscopy for metachronous lesions [12–14]. The 2021 Dutch CRC guideline similarly emphasizes CEA surveillance, despite its limited sensitivity of 50–70%, raising concerns about the ability of current protocols to reliably detect potentially curable recurrences. Although most follow-up guidelines focus on stage I–III disease, surveillance is also routinely performed in patients with stage IV CRC treated with curative intent. As evidence regarding the optimal follow-up strategy in this population remains limited and recommendations are largely extrapolated from stage I–III disease, these patients were included in the present study to reflect real-world clinical practice.

Notably, approximately 75% of patients do not develop recurrence during follow-up period. Although surveillance may provide comfort and reassurance, it also entails disadvantages such as psychological distress, false-positive test results, and increased health care costs [15]. With CRC prevalence rising, these factors contribute to a growing burden on healthcare systems.

The primary objective of this study was to identify patients with potentially curable recurrence during postoperative surveillance following curative surgery for CRC in a real‑world setting. A secondary objective was to determine the time to detection and the diagnostic modalities used, with the ultimate goal of improving surveillance strategies and minimizing unnecessary investigations.

## Methods

### Study design and population

A retrospective cohort study was performed of patients who underwent surgical treatment for stage I–IV CRC at Isala, a Dutch teaching hospital, between 1 January 2014 and 31 December 2018. Exclusion criteria were non-curable synchronous metastases, postoperative mortality, pT1N0 tumours (endoscopic follow‑up only), refusal or loss to follow‑up, and organ‑preserving treatment of the primary tumour. Patients with stage IV disease were included only if synchronous metastatic disease has been treated with curative intent and no residual disease was present at the start of surveillance. These patients were included because they are routinely enrolled in surveillance programmes in daily clinical practice despite the limited evidence base supporting follow-up in this setting.

Patients were followed for five years or until first recurrence, last contact or death. Follow‑up ended 31 December 2023. Data were accessed for research purposes between 1 January 2023 and 31 December 2023. The investigators had access to identifiable patient information during data extraction, but all data were anonymized prior to analysis.

Follow‑up adhered to the national guideline in place before the 2021 revision and consisted of serial CEA measurements, chest X‑rays, liver ultrasounds, and colonoscopies at 12 and 48 months, as outlined in Table 1. Abnormal findings prompted CT or MRI. Stage IV patients received individualized follow‑up with more frequent and advanced imaging. The majority of follow-up was performed by two dedicated nurse practitioners, contributing to a high degree of consistency in follow-up procedures.

**Table 1:** Follow-up schedule based on the Dutch guideline (2014-2021).

Months	6	12	18	24	30	36	42	48	54	60
**CEA**	X	X	X	X	X	X	X	X	X	X
**Ultrasound liver /Chest X-ray**	X	X	X	X		X		X		X
**Colonoscopy**		X						X		

Patients were categorized into three distinct groups. 1) The no-recurrence group consisted of individuals who remained disease free throughout the five-year follow-up period or who died from causes unrelated to CRC before the end of follow-up. 2) The potentially curable recurrence group included patients who developed one or more recurrences for which the multidisciplinary tumour board considered curative intent treatment feasible, including surgical resection, stereotactic ablative radiotherapy (SABR), or radiofrequency ablation (RFA). 3) The non-curable recurrence group consisted of patients whose recurrence was managed with palliative intent with systemic therapy and/or radiotherapy without a surgical resection.

The Isala Medical Ethics Review Committee confirmed that the study was not subject to the Dutch Medical Research Involving Human Subjects Act (WMO) (reference 20220905). Ethical approval was granted on 19 September 2022.

### Data collection

Primary tumour characteristics and treatment details were obtained from the Dutch ColoRectal Audit (DCRA) [16]. Five‑year follow‑up data were extracted from electronic health records, including colonoscopy findings, CEA values, postoperative complications (Clavien–Dindo), tumour stage, (neo)adjuvant therapy, the presence of synchronous metastases at presentation, recurrence characteristics, detection modality (categorised as CEA elevation, imaging without CEA elevation, or other), and survival. Time to detection was measured in months. To account for real‑world variability in scheduling, recurrence timing was additionally categorised into three-month windows around predefined follow‑up intervals. For example, the 6‑month interval included all recurrence detections between months 4 and 9. When histopathology was unavailable, multidisciplinary team conclusions served as the reference standard. Tumour staging followed the AJCC 8th edition [17]. Metachronous recurrence was defined as any recurrence after primary tumour resection. For patients receiving neoadjuvant therapy, clinical stage was used; if unavailable, pathological stage was applied.

### Statistical analysis

Categorical variables were summarised as counts and percentages; continuous variables as mean (SD) or median (IQR). Group differences were assessed using t‑tests, Mann–Whitney U tests, χ^2^ tests, or Fisher’s exact tests. Normality was evaluated with the Shapiro–Wilk test.

The primary outcome was the occurrence of a potentially curable recurrence within 60 months after resection of the primary tumour. Prognostic factors were assessed using univariate and multivariable Cox proportional hazards regression. Variables with p < 0.20 in univariate analysis were entered into the multivariable model. Collinearity was assessed using Pearson and Spearman correlations. In cases of highly correlated variables, selection was based on clinical relevance or evidence from previous studies.

As death prior to recurrence and non-curable recurrence may constitute competing events before the occurrence of a potentially curable recurrence, a Fine–Gray subdistribution hazards model was used. This approach estimates the effect of covariates or cause-specific hazards on the cumulative incidence of the event of interest, while accounting for competing risks. The cumulative incidence function (CIF) was used to quantify the marginal probability of each competing event over time. Model performance was evaluated using the concordance index (C‑index). Results are reported as hazard ratios (HR) with 95% confidence intervals (CI), and a two‑sided p‑value < 0.05 was considered statistically significant. The number needed to follow‑up (NNFU) was calculated as 1 / (potentially curable recurrences / total patients). All analyses were performed using SPSS version 29.0 (IBM Corp., Armonk, NY, USA) and Stata 19.0 (Stata Corp, Texas, USA).

## Results

### Study population

A total of 1118 patients were included in the final analysis; the study flow is depicted in Fig 1*.* The median age was 70 years (IQR 13), and one-third was aged 75 years or older. Most patients were male (55%). Stage II and III were most prevalent, representing 33% and 45% of the cohort, respectively, while 6% presented with stage IV. The primary tumour was located in the right-colon in 42% of patients, in the left colon in 29%, and in the rectum in 29%. After a follow-up of 60 months, 78% of patients (n = 868) remained free of recurrence, 11% of them died during this period from causes not related to CRC.

**Fig 1:**
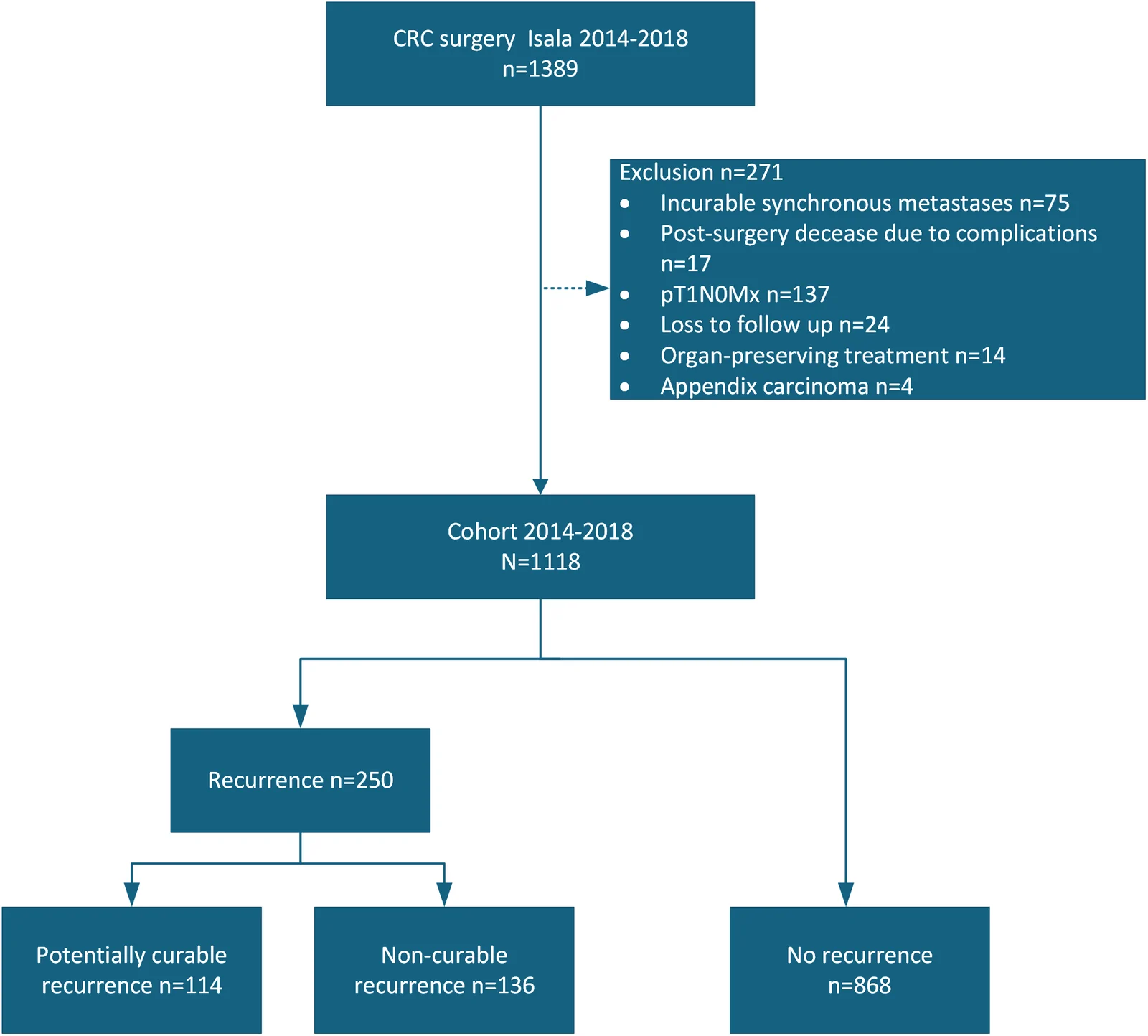
Study design and population.

### First recurrence

After 60 months of follow-up (median 60, IQR 33) 250 patients (22.4%) developed a recurrence. In addition to established pathological and histological risk factors, several additional variables were identified that were significantly associated with increased risk of recurrence: absence of complete colon visualization, (p = 0.043), CEA ≥ 5 ng/ml at diagnosis (p < 0.001), urgency of the surgical procedure (p = 0.008), and receipt of adjuvant chemotherapy (p < 0.001). Tumour localization was not statistically significant (p = 0.051), although recurrence was more frequent in rectal cancer (26%) than in colon cancer (21%). Recurrence risk increased with stage of disease stage, ranging from 6% in stage I to 13% in stage II, 28% in stage III, and 78% in stage IV. Supplement 1 provides detailed cohort characteristics and comparisons between recurrence and no‑recurrence groups.

Of the 250 patients with a recurrence, 114 (46%) were classified as having a potentially curable recurrence, and they received treatment with curative intent. In contrast, 136 patients (54%) received palliative treatment and were therefore classified as having a non-curable recurrence. Histological confirmation of a recurrence was achieved in 61% and in 87% the recurrence has been assessed in the multidisciplinary board.

Most patients (72%) presented with a single-organ recurrence, most commonly in the liver (37%), followed by the lungs (25%). Characteristics of the first recurrence, including a comparison between potentially curable and non-curable recurrences, are shown in Table 2*.* Overall, 88% of recurrences were detected during scheduled follow‑up. Imaging without CEA elevation detected 26% of all recurrences. Overall, 51% of first recurrences occurred within 12 months after primary treatment, 77% within 24 months, and 90% within 36 months.

**Table 2:** Characteristics first recurrence.

	Total	Potentially curable	Non-curable	P-value
	n=250	n=114 (46%)	n=136 (54%)	
**Detection in scheduled follow up (%)**				**0.006**
No	31 (12)	7 (6)	12 (18)	
Yes	219 (88)	107 (94)	112 (84)	
**CEA serum level ng/ml**				**0.057**
< 5	90 (37)	49 (43)	41 (31)	
≥ 5	153 (63)	64 (57)	89 (69)	
**Detection Modality**				**0.001**
Imaging	65 (26)	40 (35)	25 (18)	
CEA* (+/- imaging)	139 (56)	62 (54)	77 (57)	
Other	46 (18)	12 (11)	34 (25)	
**Single-organ**				**<0.001**
No	69 (28)	14 (12)	55 (40)	
Yes	181 (72)	100 (88)	81 (60)	
**Localization single- organ n (%) (n=181)**				
Liver	66 (37)	46 (46)	20 (25)	**0.003**
Pulmonal	46 (25)	28 (28)	18 (22)	0.375
Peritoneal / omental	29 (16)	13 (13)	16 (20)	0.218
Locoregional	24 (13)	10 (10)	14 (17)	0.151
Other	16 (9)	3 (3)	13 (16)	**0.003**
Single-organ distant versus locoregional n (%) (n=181**)				**0.039**
Distant metastases	128 (71)	77 (77)	51 (63)	
Locoregional	53 (29)	23 (23)	30 (37)	
**Mortality**				
Overall	160 (64)	41(36)	119 (87)	**<0.001**
CRC specific	157 (98)	39 (95)	118 (99)	0.162
**Time to recurrence (months)**				0.552
Median + IQR	15 (18)	16 (20)	14 (17)	

Data are expressed as n (%) unless otherwise specified.

*5 patients detected by CEA elevation but still <5 ng/ml. ** locoregional is peritoneal and locoregional, distant is liver, pulmonal and other.

Detailed baseline patient and tumour characteristics of patients with a potentially curable, a non-curable and no recurrence are described are provided in S2 Table.

### Potentially curable recurrence

Of 114 (46%) patients that received treatment with curative intent for their first recurrence, 31% experienced only one recurrence during follow-up, whereas the remaining 69% developed two to five recurrences over time, some of which were still treated with curative intent. After 60 months, 63 out of 114 patients initially curatively treated had transitioned to palliative care due to disease progression.

Sixty months after primary resection, 61 of 114 patients with a potentially curable recurrence were alive without evidence of disease, representing 5,4% of the entire cohort of 1118 patients. Twelve patients were alive while receiving palliative treatment. Of the 41 patients who had died, 39 had died from CRC, whereas two deaths were attributable to non‑CRC related causes.

Patients with a potentially curable recurrence were more likely to have rectal cancer and to have experienced postoperative complications after primary tumour resection. They had lower rates of lymfovascular invasion and N2 disease. CEA < 5 ng/mL at recurrence and single‑organ recurrence were also more common in this group. Among single‑organ recurrences, liver metastases accounted for 46% of cases and lung metastases for 28*%* (Table 2).

Among patients with a potentially curable recurrence, 94% were detected during scheduled follow‑up, compared to 84% in the non-curable group (p = 0.006). Elevated CEA levels (≥ 5 ng/mL) were present in 57% of these recurrences, which was lower than in non-curable recurrences (57% vs. 69%, p = 0.057). Pulmonary recurrences were least likely to present with elevated CEA, with only 39% showing increased levels (p < 0.0001). In both groups, the proportion of patients with CEA ≥ 5 ng/mL was higher at recurrence than at primary diagnosis (potentially curable: 46% → 57%; non-curable: 40% → 69%). Imaging without CEA elevation detected 35% of potentially curable cases, compared with 18% in the non-curable group (p = 0.001). Notably, 21 patients (18%) with a potentially curable recurrence were detected solely by imaging more than 12 months after primary treatment, compared with 14 patients (10%) in the non-curable group.

Median time to recurrence was similar between groups: 16 months (IQR 20) in the potentially curable group and 14 months (IQR 17) in the non-curable group (p = 0.522). Figure 2 visualizes these findings and shows the distribution between the two groups.

**Fig 2:**
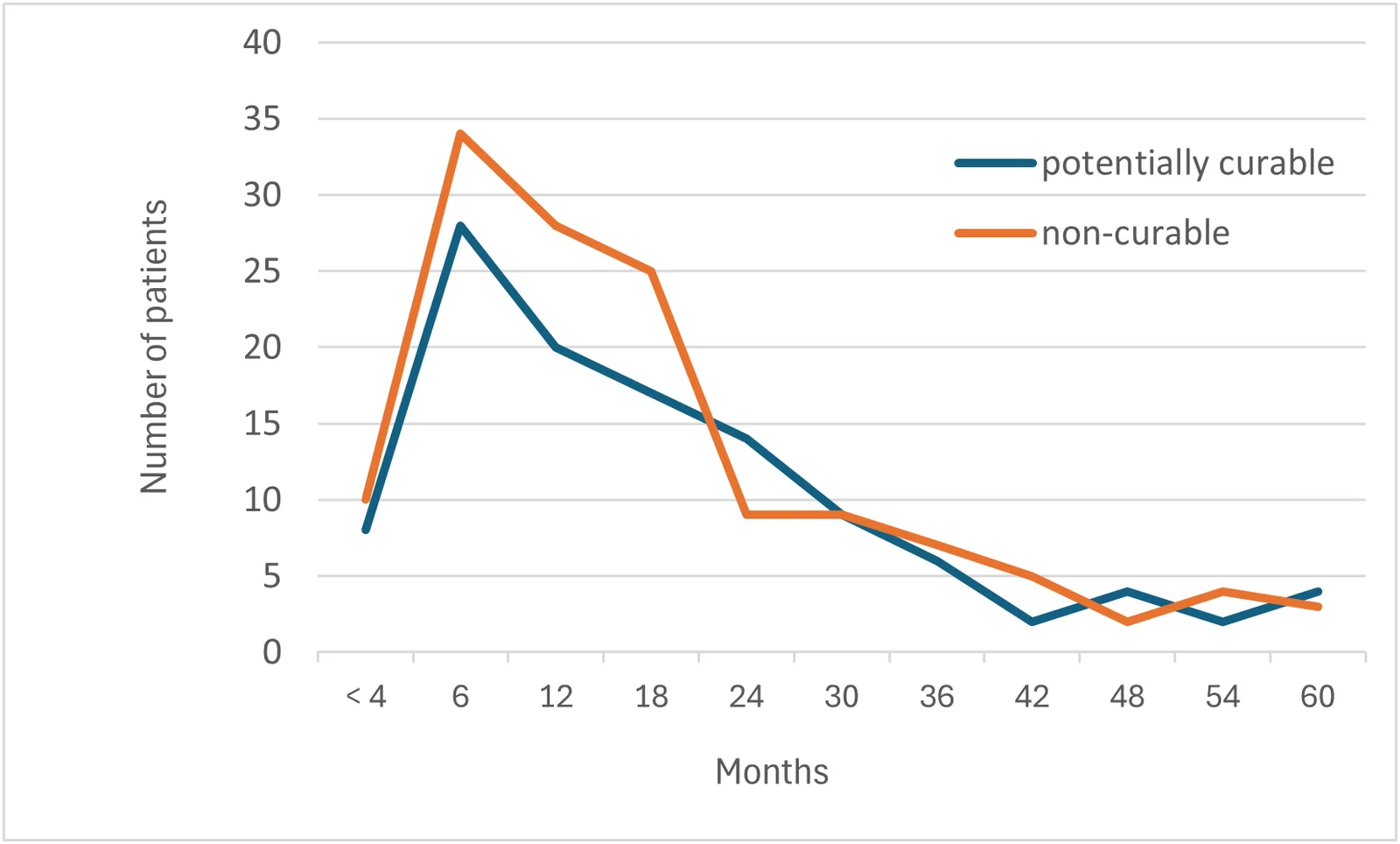
Time to detection of first recurrence in months.

### Competing risk Cox regression

Multivariable Cox proportional hazards analysis was performed on the total cohort (n = 1118) with potentially curable recurrence as outcome (n = 114). The following factors were identified as independently associated with an increased risk of a potentially curable recurrence: age (HR 0.98), postoperative minor complications (HR 1.59), lymfovascular invasion (HR 1.59), positive resection margins (HR 3.52), stage III CRC (HR 2.83) and stage IV disease (HR 15.22).

In the competing risk model, accounting for non-curable recurrence and non‑CRC mortality, age (HR 0.97), postoperative minor complications (HR 1.78), stage III CRC (HR 2.76) and stage IV disease (HR 11.02) were significant associated with a potentially curable recurrence. Detailed hazard ratios from both models are presented in Table 3. At 60 months, the cumulative incidence functions (CIF) for potentially curable recurrence, non-curable recurrence, and non-CRC death were 8.0%, 8.8%, and 4.8%, respectively. The final model demonstrated moderate discriminative performance, with a C-index of 0.75.

**Table 3:** Univariate and multivariate Cox regression and competing risk Cox models of variables associated with potentially curable CRC recurrence.

Prognostic factor	Univariable Cox regression HR (CI 95%)	P-value	Multivariable Cox regression HR (CI 95%)	P-value	Competing risk Cox-model SHR (CI 95%)	P-value
**Age at time of surgery**	0.98 (0.96-0.99)	**0.006**	0.98 (0.96-1.00)	**0.048**	0.97 (0.96-0.99)	**0.006**
**Gender**						
Male	ref		ref		ref	
Female	0.75 (0.52-1.10)	0.144	0.88 (0.59-1.30)	0.535	0.84 (0.56-1.25)	0.398
**Complete colon visualization**						
No	ref					
Yes	0.81 (0.53-1.21)	0.300				
**CEA at primary diagnosis CRC**						
< 5 ng/ml	ref		ref		ref	
≥ 5 ng/ml	2.07 (1.42-3.02)	**<0.001**	1.43 (0.96-2.13)	0.077	1.35 (0.91-2.02)	0.131
**Localization primary tumour**						
Colon	ref		ref			
Rectum	1.74 (1.20-2.52)	**0.004**	1.24 (0.80-1.91)	0.335		
**Urgency of procedure**						
Elective	ref					
Urgent	1.41 (0.74-2.71)	0.295				
**Complications after surgery**						
No complications	ref		ref		ref	
Minor complications (Clavien-Dindo 1–2)	1.83 (1.24-2.70)	**0.002**	1.59 (1.05-2.43)	**0.030**	1.78 (1.16-2.72)	**0.008**
Major complications (Clavien-Dindo ≥ 3)	1.50 (0.82-2.76)	**0.191**	1.12 (0.59-2.11)	0.726	1.12 (0.61-2.08)	0.703
**Tumour differentation**						
Good/moderate	ref					
Poor/ undifferentiated	0.85 (0.47-1.55)	0.594				
**Lymfovascular invasion**						
Absent	ref		ref		ref	
Present	2.30 (1.58-3.33)	**<0.001**	1.59 (1.04-2.42)	**0.033**	1.33 (0.87-2.02)	0.191
**Resection margin**						
R0	ref		ref		ref	
R1	5.06 (2.06-12.42)	**<0.001**	3.52 (1.38-9.00)	**0.009**	2.26 (0.80-6.33)	0.122
**T-stage**						
pT0	ref					
pT1	1.66 (0.28-9.93)	0.579				
pT2	0.88 (0.20-3.84)	0.867				
pT3	1.82 (0.45-7.43)	0.403				
pT4	3.61 (0.85-15.29)	0.081				
**M-stage**						
M0	ref					
M1	8.71 (5.5-13.75)	**<0.001**				
**Adjuvant chemotherapy**						
No	ref					
Yes	1.48 (1.00-2.21)	0.052				
**Tumour-stage**						
I	ref		ref		ref	
II	2.14 (0.87-5.24)	0.095	1.99 (0.80-4.91)	0.138	1.84 (0.77-4.44)	0.173
III	4.07 (1.75-9.43)	**0.001**	2.83 (1.19-6.73)	**0.019**	2.76 (1.19-6.38)	**0.018**
IV	26.30 (10.81-63.96)	**<0.001**	15.22 (8.98-38.74)	**<0.001**	11.02 (4.44- 27.37)	**0.000**

Numbers of patients per outcome category were potentially curable (n = 114), non‑curable (n = 136), and death of non-CRC cause (n = 96).

### Numbers needed to follow-up

The overall number needed to follow‑up (NNFU) was 9.8, calculated as 1 / (114/1118). This NNFU indicates that approximately ten patients must be followed to detect one potentially curable recurrence. NNFU varied substantially by tumour location and stage (Table 4).

**Table 4:** Numbers needed to follow-up (NNFU) for potentially curable recurrence per localization primary tumour and disease stage.

	Total	Recurrence	Potentially curable recurrence	NNFU
**Total**	1118	250 (22)	114 (10)	**9.8**
**Colon**	793	165 (21)	67 (8)	**11.8**
RCC	467	91 (19)	34 (7)	**13.7**
LCC	326	74 (23)	33 (10)	**9.9**
**Rectum**	325	85 (26)	47 (14)	**6.9**
**Stage I**	182	11 (6)	6 (3)	**30.3**
**Stage II**	373	48 (13)	24 (6)	**15.5**
**Stage III**	500	142 (28)	57 (11)	**8.8**
**Stage IV**	63	49 (78)	27 (43)	**2.3**

Data are expressed as n (%). RCC; right-sided colon cancer, LCC; left-sided colon cancer.

Across tumour sites, values ranged from 6.9 to 13.7. Right‑sided colon cancer had the lowest rate of potentially curable recurrence (7%), resulting in the highest NNFU (13.7). Rectal cancer had the highest rate (14%), corresponding to the lowest NNFU (6.9). When stratified by stage, Stage I patients had the lowest potentially curable recurrence rate (3.0%), leading to an NNFU of 30.3. Conversely, Stage IV patients exhibited a potentially curable recurrence rate of 43%, corresponding to an NNFU of 2.3. As shown in Fig 3, combining tumour location and stage demonstrates that rectal cancer consistently requires fewer patients to be followed to detect one potentially curable recurrence, with the largest difference observed in stage II disease.

**Fig 3:**
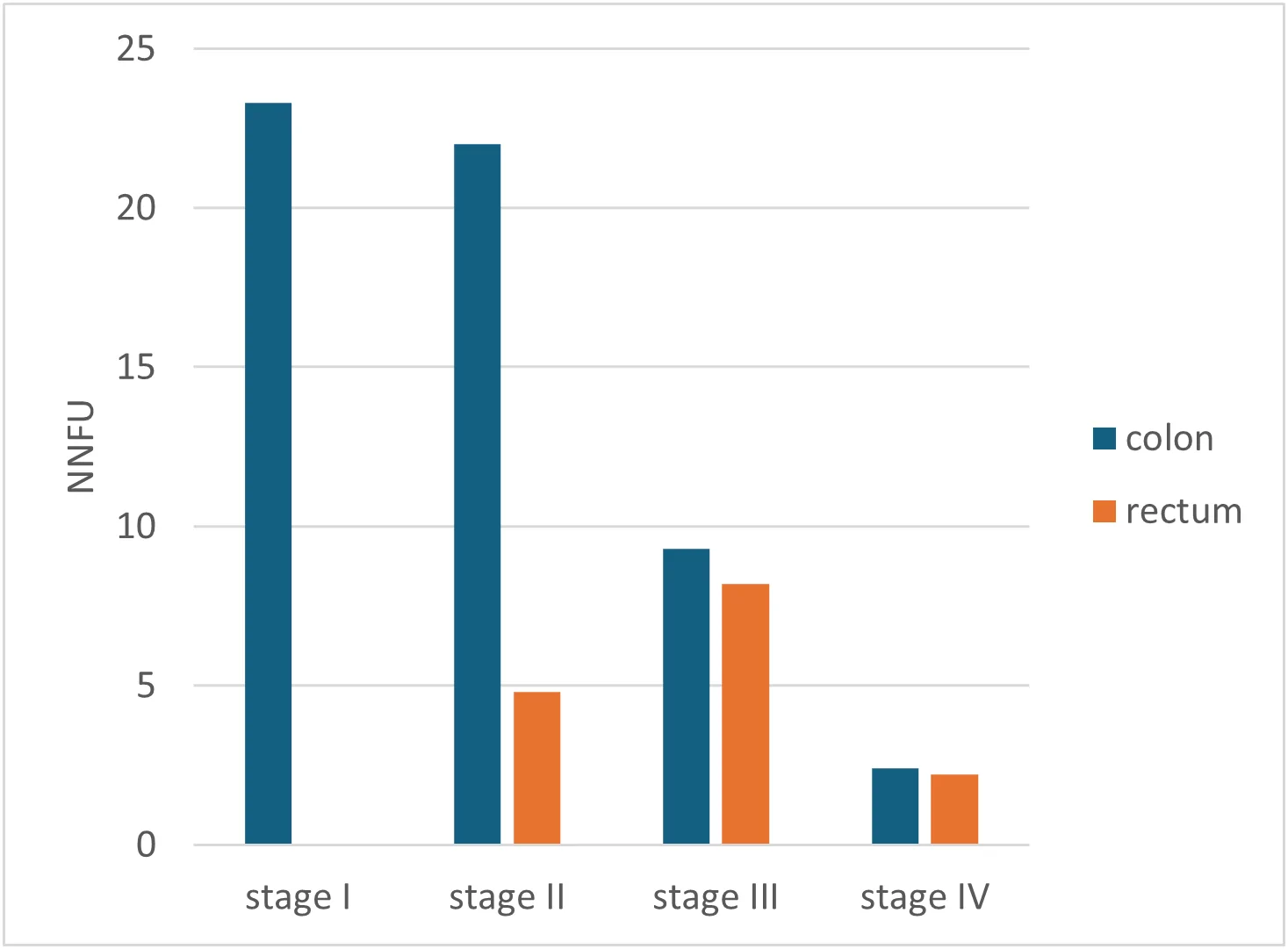
Number needed to follow-up (NNFU) for potentially curable recurrence for colon and rectal cancer by stage.

## Discussion

This study evaluated the frequency and characteristics of potentially curable recurrences after curative resection of stage I–IV CRC in a real‑world hospital setting. After a median follow up of 60 months, recurrence occurred in 22.4% of patients, and the CIF of potentially curable recurrence was 8.0%. Independent predictors included age, postoperative minor complications, and stage III and IV disease. Potentially curable recurrences were more often asymptomatic, had normal CEA levels (<5 ng/ml), were limited to a single organ, and occurred more frequently in the liver compared with palliatively treated recurrences.

These recurrence rates are consistent with previous studies [4,18–20]. The FACS trial reported recurrence in 16.6% of stage I–III patients, and the COLOFOL trial observed a 21.4% in stage II–III in patients aged ≤ 75 years. Although our real‑world cohort included stage IV patients, in case of excluding them the 5‑year recurrence rate was 19%.

The 60‑month CIF of 8.0% reflects the probability of detecting a potentially curable recurrence while accounting for the competing risks of non-curable recurrence and non‑CRC death. The moderate discriminative performance of our model (C‑index 0.75) indicates that the included predictors provide meaningful risk stratification although improvement is possible. Recent literature highlights several biological and treatment‑related factors that may enhance model performance but could not be reliably incorporated in our study due to inconsistent reporting or insufficient data quality. For example, vascular and perineural invasion have been identified as important predictors of recurrence [21]. In our study, lymphatic and vascular invasion were combined into a single variable due to inconsistent reporting, this approach may underestimate the predictive value of vascular invasion specifically. In rectal cancer, perineural invasion has been shown to strongly predict local recurrence, while EMVI on MRI is associated with an increased risk of distant recurrence [21]. Similarly, Boute et al., reported that extramural vascular invasion was the strongest predictor of recurrence among patients with primary rectal cancer, substantially outperforming lymphatic invasion [18].

Emerging biomarkers such as circulating tumour DNA (ctDNA) further hold promise for improving recurrence prediction [22,23]. However, despite increasing evidence for the prognostic value of ctDNA, its role in routine surveillance and its impact on long-term survival remain under investigation.

The observed association between minor postoperative complications and potentially curable recurrence was unexpected, as postoperative morbidity is generally associated with poorer oncological outcomes. Previous studies have reported inferior survival following major complications (Clavien-Dindo 3–4) [24]. In the present competing-risks analysis, however, only minor complications were independently associated with a potentially curable recurrence, whereas no such association was observed for major complications. This finding is unlikely to reflect a beneficial effect of postoperative complications themselves. Rather, it may be explained by differences in surveillance intensity, earlier recurrence detection, or competing-risk mechanisms that affect the likelihood of detecting recurrence at a potentially curable stage. The absence of an association for major complications may be related to limited statistical power or differences in recurrence patterns among these patients. Further research is warranted to better understand the mechanisms underlying this association.

Although 88% of recurrences were detected during scheduled follow‑up, 43% of potentially curable recurrences occurred with a normal CEA level. Pulmonary recurrences often lacked CEA elevation. This aligns with prior evidence showing that CEA has a sensitivity of only 50–70% for detecting recurrence [25,26]. The Dutch CRC guideline revision of 2021, which recommended CEA after the 12‑month CT scan, may therefore miss potentially curable recurrences [12]. It remains uncertain whether CEA levels in these patients would eventually exceed the threshold, and whether such delays would compromise curative outcomes. The CEA Watch study reported that 80% of recurrences were associated with CEA elevation, though a rise was defined liberally, potentially inflating sensitivity at the expense of specificity [6]. While current clinical practice does incorporate CEA kinetics over time to improve sensitivity, our study did not apply this approach, representing a limitation. These findings underscore the importance of dynamic monitoring strategies, where the rise and doubling time of CEA are emphasized over absolute values. Although CEA testing offers advantages such as low cost, feasibility in community settings, and integration with eHealth platforms, its limited sensitivity remains a challenge for an effective follow-up.

A key finding of this study is the substantial variation in the NNFU across tumour localizations and stages. The NNFU ranged from 2.3 in stage IV to 30.3 in stage I, and from 6.9 in rectal cancer to 13.7 in right‑sided colon cancer. These differences reflect underlying biological heterogeneity and are consistent with earlier observations that recurrence risk and curability differ significantly between subgroups [26]. The findings strengthen the argument for a more individualized follow-up strategy and align with the current Dutch CRC guideline, which allows omission of follow‑up in low‑risk stage I patients. The low yield of stage I patients suggests limited clinical benefit of routine follow-up, while contributing to unnecessary diagnostic procedures, patient burden, and healthcare costs.

### Strengths & limitations

This study has several strengths. It was conducted within a single institution using a uniform follow-up protocol, minimizing procedural variability. The use of real-world data (RWD) reflects daily clinical practice and capture the complexity of patient trajectories beyond the constraints of randomized trials. A particular strength is the inclusion of patients with stage IV CRC treated with curative intent. Although these patients are often underrepresented in surveillance studies and are not the primary focus of most follow-up guidelines, they are frequently monitored in routine practice, while evidence to guide optimal surveillance remains limited. Their inclusion therefore enhances the real-world applicability and generalizability of our findings.

The importance of RWD is underscored by recent findings from Elferink et al. (2025), who reported a median overall survival of 16 months for metastatic colorectal cancer in routine practice in the Netherlands. This is substantially lower than the 30 months reported in selected populations [27]. This discrepancy underscores the need for studies that reflect actual treatment realities and outcomes, rather than idealized trial conditions.

The availability of curative treatment options for metastatic recurrence—such as liver resections, radiofrequency ablation (RFA), and stereotactic ablative radiotherapy (SABR)—within our centre is both a strength and a limitation. It enables early intervention and realistic evaluation of outcomes but may also introduce bias by influencing follow-up intensity and recurrence detection, potentially limiting generalizability to settings with fewer therapeutic resources. A further limitation is the retrospective design of the study.

### Clinical implications

Follow‑up should be tailored to individual recurrence risk and patient preferences. At the start of surveillance, clinicians should discuss prognosis, expected benefit, and patient goals. Recurrence itself represents a second major decision point, requiring reassessment of prognosis and patient wishes. Surveillance effectiveness differs substantially among subgroups, with greatest benefit in rectal cancer and stage IV disease.

### Conclusion

Standard follow-up enabled the detection of potentially curable recurrence in 8% of CRC patients. The substantial variation in NNFU across subgroups and the limited sensitivity of CEA highlight the need for more personalized, risk‑adapted surveillance. Aligning follow‑up intensity with individual recurrence risk may improve detection of treatable recurrences while reducing unnecessary investigations.

## Supporting information

S1 Table Baseline patient and tumour characteristics. (DOCX)

S2 Table Baseline patient and tumour characteristics of patients with a potentially curative, a non-curable and no recurrence. (DOCX)

## Abbreviations

AJCC: American Joint Committee on Cancer
CEA: Carcino-Embryonic Antigen
CI: confidence interval
CIF: Cumulative incidence function
CRC: Colorectal cancer
DCRA: Dutch ColoRectal Audit
HR: hazard ratio
NNFU: number needed to follow-up
RFA: radiofrequency ablation
SABR: stereotactic ablative radiotherapy.
