# Supplementary material for: Detection of potentially curable recurrence after colorectal cancer surgery in a real-world population
Source: PLoS One. 2026 Oct 30;21(10):e0361933. doi: 10.1371/journal.pone.0361933 (PMC13722166; doi:10.1371/journal.pone.0361933)
Supplement: Supplementary file 1 [file pone.0361933.s001.docx]

#### **S1 Table. Baseline patient and tumour characteristics.**

|  | Total | Recurrence | No recurrence | Missing | | P-value | |
| --- | --- | --- | --- | --- | --- | --- | --- |
| Numbers n (%) | n=1118 | n=250 | n=868 |  |  | |  |
| Age at time of surgery |  |  |  | 0 | 0.150 | |  |
| Mean ± SD | 69 (11) | 68 (11) | 69 (10) |  |  | |  |
| Median met IQR | 70 (13) | 70 (12) | 70 (13) |  |  | |  |
| Gender |  |  |  | 0 | 0.215 | |  |
| Male | 610 (55) | 145 (58) | 465 (53) |  |  | |  |
| Female | 508 (45) | 105 (42) | 403 (46) |  |  | |  |
| ASA 3 classes |  |  |  | 0 | 0.582 | |  |
| I | 244 (22) | 56 (22) | 188 (22) |  |  | |  |
| II | 681 (61) | 146 (58) | 535 (62) |  |  | |  |
| ≥ III | 193 (17) | 48 (19) | 145 (17) |  |  | |  |
| Preoperative |  |  |  |  |  | |  |
| Complete colon visualization |  |  |  | 81 | **0.043** | |  |
| No | 312 (30) | 80 (36) | 232 (29) |  |  | |  |
| Yes | 725 (70) | 145 (64) | 580 (71) |  |  | |  |
| Blood transfusion peri-operative |  |  |  | 0 | 0.540 | |  |
| No | 944 (84) | 208 (83) | 736 (85) |  |  | |  |
| Yes | 174 (16) | 42 (17) | 132 (15) |  |  | |  |
| CEA serum level at diagnosis ng/ml |  |  |  | 57 | **< 0.001** | |  |
| < 5 | 718 (68) | 136 (57) | 582 (71) |  |  | |  |
| ≥ 5 | 343 (32) | 102 (43) | 241 (29) |  |  | |  |
| Localization primary tumour |  |  |  | 0 | 0.051 | |  |
| Colon | 793 (71) | 165 (66) | 628 (72) |  |  | |  |
| *RCC* | *467 (42)* | *91 (36)* | *376 (43)* |  |  | |  |
| *LCC* | *326 (29)* | *74 (30)* | *252 (29)* |  |  | |  |
| Rectal | 325 (29) | 85 (34) | 240 (28) |  |  | |  |
| Admission |  |  |  |  |  | |  |
| Urgency of procedure |  |  |  | 0 | **0.008** | |  |
| Elective | 1036 (93) | 222 (89) | 814 (94) |  |  | |  |
| Urgent | 82 (7) | 28 (11) | 54 (6) |  |  | |  |
| Complications after surgery |  |  |  | 0 | 0.078 | |  |
| No | 649 (58) | 133 (53) | 516 (59) |  |  | |  |
| Yes | 469 (42) | 117 (47) | 352 (41) |  |  | |  |
| Clavien-dindo |  |  |  | 0 | 0.200 | |  |
| 0 | 647 (58) | 133 (53) | 514 (59) |  |  | |  |
| 1 | 57 (5) | 17 (7) | 40 (5) |  |  | |  |
| 2 | 292 (26) | 67 (27) | 225 (26) |  |  | |  |
| ≥ 3 | 122 (11) | 33 (13) | 89 (10) |  |  | |  |
| Pathology |  |  |  |  |  | |  |
| Histological type |  |  |  | 0 | **0.003** | |  |
| Adeno | 1003 (90) | 216 (86) | 787 (91) |  |  | |  |
| Mucinous | 104 (9) | 27 (11) | 77 (9) |  |  | |  |
| Signet cell | 11 (1) | 7 (3) | 4 (1) |  |  | |  |
| Differentiation grade |  |  |  | 56 | **0.049** | |  |
| Good/moderate | 922 (87) | 201 (83) | 721 (88) |  |  | |  |
| Poor | 140 (13) | 41 (17) | 99 (12) |  |  | |  |
| Lymfovascular |  |  |  | 0 | **<0.001** | |  |
| No | 811 (73) | 128 (51) | 683 (79) |  |  | |  |
| Yes | 307 (27) | 122 (49) | 185 (21) |  |  | |  |
| Resection margin |  |  |  | 0 | **<0.001** | |  |
| R0 | 1100 (98) | 238 (95) | 862 (99) |  |  | |  |
| ≥R1 | 18 (2) | 12 (5) | 6 (1) |  |  | |  |
| T-stage |  |  |  | 0 | **<0.001** | |  |
| pT0 | 30 (3) | 3 (1) | 27 (3) |  |  | |  |
| pT1 | 28 (3) | 5 (2) | 23 (3) |  |  | |  |
| pT2 | 278 (25) | 29 (12) | 249 (29) |  |  | |  |
| pT3 | 641 (57) | 150 (60) | 491 (57) |  |  | |  |
| pT4 | 141 (13) | 63 (25) | 78 (9) |  |  | |  |
| N-stage |  |  |  | 0 | **<0.001a** | |  |
| pN0 | 693 (62) | 88 (35) | 605 (70) |  |  | |  |
| pN1 | 288 (26) | 93 (37) | 195 (23) |  |  | |  |
| pN2 | 137 (12) | 69 (28) | 68 (8) |  |  | |  |
| M-stage |  |  |  | 0 | **<0.001** | |  |
| M0 | 1061 (95) | 206 (82) | 855 (99) |  |  | |  |
| M1 | 57 (5) | 44 (18) | 13 (1) |  |  | |  |
| Adjuvant chemotherapy |  |  |  | 0 | **<0.001** | |  |
| No | 858 (77) | 165 (66) | 693 (80) |  |  | |  |
| Yes | 260 (23) | 85 (34) | 175 (20) |  |  | |  |
| Tumour Stage * |  |  |  | 0 | **<0.001** | |  |
| I | 182 (16) | 11 (4) | 171 (20) |  |  | |  |
| II | 373 (33) | 48 (19) | 325 (37) |  |  | |  |
| III | 500 (45) | 142 (57) | 358 (41) |  |  | |  |
| IV | 63 (6) | 49 (20) | 14 (2) |  |  | |  |
| Mortality |  |  |  | 0 |  | |  |
| Overall | 256(23) | 160 (64) | 96 (11) |  | <0.001 | |  |
| CRC specific | 157 (61) | 157 (98) | 0 |  | <0.001 | |  |

*Data are expressed as n (%) unless otherwise specified. ASA;American Society of Anesthesiologists; Location primary tumour; RCC;Right-sided colon cancer, coecum to splenic flexure, LCC; Left-sided colon cancer, splenic flexure to rectum, included recto-sigmoid, RC; rectal cancer. Note: * For ypTNM stage, the corresponding cTNM stage was used*
