# Supplementary material for: Detection of potentially curable recurrence after colorectal cancer surgery in a real-world population
Source: PLoS One. 2026 Oct 30;21(10):e0361933. doi: 10.1371/journal.pone.0361933 (PMC13722166; doi:10.1371/journal.pone.0361933)
Supplement: Supplementary file 2 [file pone.0361933.s002.docx]

**S2 Table*.* Baseline patient and tumour characteristics of patients with a potentially curative, a non-curable and no recurrence.**

|  | Total | Potentially curable | Non-curable | No recurrence | P-value ^A^ | P-value ^B^ | P- value ^C^ |
| --- | --- | --- | --- | --- | --- | --- | --- |
| Numbers n (%) | N=1118 | N= 114 | N=136 | N=868 |  |  |  |
| Age at time of surgery |  |  |  |  | **0.002** | **0.001** | 0.376 |
| Mean ± SD | 69 (11) | 66 (11) | 70 (11) | 69 (10) |  |  |  |
| Median met IQR | 70 (13) | 68 (13) | 73 (11) | 70 (13) |  |  |  |
| Gender n (%) |  |  |  |  | 0.318 | 0.114 | 0.732 |
| Male | 610 (55) | 70 (61) | 75 (55) | 465 (54) |  |  |  |
| Female | 508 (45) | 44 (39) | 61 (45) | 403 (46) |  |  |  |
| ASA 3 classes |  |  |  |  | 0.540 | 0.593 | 0.524 |
| I | 244 (22) | 29 (25) | 27 (20) | 188 (22) |  |  |  |
| II | 681 (61) | 65 (57) | 81 (60) | 535 (62) |  |  |  |
| ≥ III | 193 (17) | 20 (17) | 28 (21) | 145 (17) |  |  |  |
| Preoperative |  |  |  |  |  |  |  |
| Complete colon visualization |  |  |  |  | 0.406 | 0.383 | **0.034** |
| No | 312 (30) | 34 (33) | 46 (38) | 232 (29) |  |  |  |
| Yes | 725 (70) | 70 (67) | 75 (62) | 580 (71) |  |  |  |
| Blood transfusion  peri-operative |  |  |  |  | 0.284 | 0.742 | 0.244 |
| No | 944 (84) | 98 (86) | 110 (81) | 736 (85) |  |  |  |
| Yes | 174 (16) | 16 (14) | 26 (19) | 132 (15) |  |  |  |
| CEA serum level at diagnosis ng/ml |  |  |  |  | 0.388 | **<0.001** | **0.012** |
| < 5 | 718 (68) | 59 (54) | 77 (60) | 582 (71) |  |  |  |
| ≥ 5 | 343 (32) | 50 (46) | 52 (40) | 241 (29) |  |  |  |
| Localization |  |  |  |  | 0.057 | **0.005** | 0.948 |
| RCC | 467 (42) | 34 (30) | 57 (42) | 376 (43) |  |  |  |
| LCC | 326 (29) | 33 (29) | 41 (30) | 252 (29) |  |  |  |
| Rectal | 325 (29) | 47 (41) | 38 (28) | 240 (28) |  |  |  |
| Localization |  |  |  |  | **0.027** | **0.003** | 0.944 |
| Colon | 793 (71) | 67 (59) | 98 (72) | 628 (72) |  |  |  |
| Rectal | 325 (29) | 47 (41) | 38 (28) | 240 (28) |  |  |  |
| Admission |  |  |  |  |  |  |  |
| Urgency of procedure |  |  |  |  | 0.265 | 0.300 | **0.003** |
| Elective | 1036 (93) | 104 (91) | 118 (87) | 814 (94) |  |  |  |
| Urgent | 82 (7) | 10 (9) | 18 (13) | 54 (6) |  |  |  |
| Complications after surgery |  |  |  |  | **0.028** | **0.005** | 0.980 |
| No | 649 (58) | 52 (46) | 81 (60) | 516 (59) |  |  |  |
| Yes | 469 (42) | 62 (54) | 55 (40) | 352 (41) |  |  |  |
| Clavien-dindo |  |  |  |  | **0.035** | **0.016** | 0.370 |
| 0 | 647 (58) | 52 (46) | 81 (60) | 514 (59) |  |  |  |
| 1 | 57 (5) | 11 (10) | 6 (4) | 40 (5) |  |  |  |
| 2 | 292 (26) | 38 (33) | 29 (21) | 225 (26) |  |  |  |
| ≥ 3 | 122 (11) | 13 (11) | 20 (15) | 89 (10) |  |  |  |
| Pathology |  |  |  |  |  |  |  |
| Histological type |  |  |  |  | 0.242 | 0.238 | **<0.001** |
| Adeno | 1003 (90) | 103 (90) | 113 (83) | 787 (91) |  |  |  |
| Mucinous | 104 (9) | 9 (8) | 18 (13) | 77 (9) |  |  |  |
| Signet cell | 11 (1) | 2 (2) | 5 (4) | 4 (1) |  |  |  |
| Differentiation grade |  |  |  |  | **0.019** | 0.700 | **0.002** |
| Good/moderate | 922 (87) | 99 (89) | 102 (78) | 721 (88) |  |  |  |
| Poor | 140 (13) | 12 (11) | 29 (22) | 99 (12) |  |  |  |
| Lymfovascular invasion |  |  |  |  | 0.053 | **<0.001** | **<0.001** |
| No | 811 (72) | 66 (58) | 62 (46) | 683 (79) |  |  |  |
| Yes | 307 (27) | 48 (42) | 74 (54) | 185 (21) |  |  |  |
| Resection margin |  |  |  |  | 0.779 | **0.005** | **<0.001** |
| R0 | 1100 (98) | 109 (96) | 129 (95) | 862 (99) |  |  |  |
| ≥R1 | 18 (2) | 5 (4) | 7 (5) | 6 (1) |  |  |  |
| T-stage |  |  |  |  | 0.483 | **<0.001** | **<0.001** |
| pT0 | 30 (3) | 2 (2) | 1 (1) | 27 (3) |  |  |  |
| pT1 | 28 (3) | 3 (43 | 2 (1) | 23 (3) |  |  |  |
| pT2 | 278 (25) | 16 (14) | 13 (10) | 249 (29) |  |  |  |
| pT3 | 641 (57) | 69 (61) | 81 (60) | 491 (57) |  |  |  |
| pT4 | 141 (13) | 24 (21) | 39 (29) | 78 (9) |  |  |  |
| N-stage |  |  |  |  | **0.054** | **<0.001** | **<0.001** |
| pN0 | 693 (62) | 45 (39) | 43 (32) | 605 (70) |  |  |  |
| pN1 | 288 (26) | 46 (40) | 47 (35) | 195 (23) |  |  |  |
| pN2 | 137 (12) | 23 (20) | 46 (34) | 68 (8) |  |  |  |
| M-stage |  |  |  |  | 0.189 | **<0.001** | **<0.001** |
| M0 | 1061 (95) | 90 (79) | 116 (85) | 855 (99) |  |  |  |
| M1 | 57 (5) | 24 (21) | 20 (15) | 13 (1) |  |  |  |
| Adjuvant chemotherapy |  |  |  |  | 0.313 | **0.010** | **<0.001** |
| No | 858 (77) | 79 (69) | 86 (63) | 693 (80) |  |  |  |
| Yes | 260 (23) | 35 (31) | 50 (37) | 175(20) |  |  |  |
| Tumour stage * |  |  |  |  | 0.239 | **<0.001** | **<0.001** |
| I | 182 (16) | 6 (5) | 5 (4) | 171 (20) |  |  |  |
| II | 373 (33) | 24 (21) | 24 (18) | 325 (37) |  |  |  |
| III | 500 (45) | 57 (50) | 85 (63) | 358 (41) |  |  |  |
| IV | 63 (6) | 27 (24) | 22 (16) | 14 (2) |  |  |  |
| Mortality |  |  |  |  |  |  |  |
| Overall | 256 (23) | 41 (36) | 119 (87) | 96 (11) | **<0.001** | **<0.001** | **<0.001** |
| CRC specific | 194 (61) | 39 (95) | 118 (99) | 0 | 0.162 | **<0.001** | **<0.001** |

*Data are expressed as n (%) unless otherwise specified ASA;American Society of Anesthesiologists; Location primary tumour; RCC;Right-sided colon cancer, coecum to splenic flexure, LCC; Left-sided colon cancer, splenic flexure to rectum, included recto-sigmoid, RC; rectal cancer. Note: * for ypTNM stage the corresponding cTNM was used*

A. *difference between patients with a potentially curable or a non-curable recurrence*
B. *difference between patients with a potentially curable or no recurrence*
C. *difference between patients with a non-curable or no recurrence*
