# Supplementary material for: Detection of potentially curable recurrence after colorectal cancer surgery in a real-world population
Source: PLoS One. 2026 Oct 30;21(10):e0361933. doi: 10.1371/journal.pone.0361933 (PMC13722166; doi:10.1371/journal.pone.0361933)
Supplement: Supplementary file 4 [file pone.0361933.s005.docx]

Octobre 01, 2026

PONE-D-26-09874; **Detection of potentially curable recurrence after colorectal cancer surgery in a real-world population**

Dear editor and reviewers,

We would like to sincerely thank you for your careful re-evaluation of our manuscript and for your constructive feedback. We are pleased that only a few minor issues remained to be addressed. We have carefully considered both comments and revised the manuscript accordingly. Detailed responses to each comment are provided below.

Sincerely,

Ivonne Schoenaker

**Comment 1.** Cumulative incidence functions for the competing events non-curable recurrence and non-CRC death.

We thank the editor and reviewer for this insightful comment. We agree that, as stated in the methods section, cumulative incidence functions should be reported for all competing events. We have therefore added the cumulative incidence functions estimates for non-curable recurrence and non-CRC death to the results section.

The updated cumulative incidence estimates were 8.0% for potentially curable recurrence (n=114), 8.8% for non-curable recurrence (n=136), and 4.8% for non-CRC death (n=96). We have added the following sentence to the Results section: *At 60 months, the cumulative incidence functions of potentially curable recurrence, non-curable recurrence, and non-CRC death were 8.0%, 8.8%, and 4.8%, respectively*

During revision of the manuscript, we identified that non-CRC mortality had inadvertently not been included as a competing event in the original competing-risk analyses. Consequently, we repeated both the cumulative incidence function analyses and the competing-risk Cox regression analyses including non-CRC mortality as intended.

This resulted in only minimal changes in the estimated subdistribution hazard ratios and corresponding confidence intervals. The only notable difference was observed for resection margin, for which the p-value changed from 0.047 to 0.122. These revisions have been incorporated into the manuscript and table 3.

**Comment 2** Interpretation of “direct therapeutic benefit in the conclusion

We agree that the previous wording could be interpreted as implying a causal therapeutic benefit attributable to surveillance. To avoid overinterpretation, we have revised the conclusion and now state that standard follow-up enabled the detection of potentially curable recurrence in 8% of CRC patients.
