# Supplementary material for: Detection of potentially curable recurrence after colorectal cancer surgery in a real-world population
Source: PLoS One. 2026 Oct 30;21(10):e0361933. doi: 10.1371/journal.pone.0361933 (PMC13722166; doi:10.1371/journal.pone.0361933)
Supplement: Supplementary file 5 [file pone.0361933.s004.docx]

August 11, 2026

PONE-D-26-09874; **Detection of potentially curable recurrence after colorectal cancer surgery in a real-world population**

Dear editor and reviewer,

Thank you for the opportunity to revise our manuscript “Detection of potentially curable recurrence after colorectal cancer surgery in a real-world population”. We appreciate the reviewers’ thoughtful comments and suggestions, which have helped us to improve the clarity and rigor of our work.

Below, we provide a detailed point-by-point response to each comment. We have addressed all concerns and revised the manuscript accordingly. Changes are incorporated in the file using track changes.

Sincerely,

Ivonne Schoenaker

**Journal Requirements:**

**1. Regarding PLOS ONE's style requirements**

**Answer**: *We have reviewed the manuscript and associated files to ensure compliance with the PLOS ONE formatting and file naming requirements. The manuscript formatting has been checked, and minor adjustments have been made where applicable.*

**2. Regarding the Data Availability Statement.**

**Answer:** *The study contains detailed individual patient-level clinical information from a retrospective single-centre cohort study (Isala Hospital). Although direct identifiers have been removed, the dataset still contains both direct event-related and indirect identifiers that create a substantial risk of patient re-identification, particularly given the relatively small study population.*

*According to the BMJ guidance on preparing raw clinical data for publication (Hrynaszkiewicz et al., BMJ 2010), anonymization should ensure that individuals cannot reasonably be re-identified from the combination of variables provided. Variables that may enable re-identification include:*

- *Dates of surgery;*
- *Dates of recurrence events;*
- *Dates of death;*
- *Place of treatment (single-centre study conducted at Isala Hospital, Zwolle the Netherlands));*
- *Sex;*
- *Disease characteristics;*
- *Year of birth.*

*Because these variables are essential for the analyses and outcomes reported in the manuscript, their removal or extensive aggregation would prevent meaningful replication of the study findings.*

*Due to privacy restrictions and the sensitive nature of the patient data, the dataset cannot be made publicly available. The study data are stored locally in the Research Manager Electronic Data Capture (EDC) system of Isala and will be retained in accordance with institutional data management and retention policies.*

*Requests for access to the data from qualified researchers may be submitted to the Isala Research Office via* ***Onderzoek@Isala-Academie.nl****. This institutional contact point is independent of the authors and will coordinate and evaluate data access requests in accordance with applicable ethical, legal, and privacy regulations.*

*We therefore propose the following Data Availability Statement:*

*The data underlying the results presented in this study cannot be shared publicly because they contain sensitive patient information and are subject to privacy regulations. The data are stored within the Research Manager Electronic Data Capture (EDC) system of Isala. Qualified researchers may request access to the data by contacting the Isala Research Office at Onderzoek@Isala-Academie.nl. Requests will be assessed in accordance with applicable ethical, legal, and privacy requirements. Data will be retained and managed according to institutional data management policies*

**3) Inclusion of stage IV patients**

**Answer:** *We thank the Editor for this comment. As detailed in our response to Reviewer 1, the inclusion of stage IV patients treated with curative intent surgery was considered a strength of the study because it reflects routine clinical practice and enhances the real-world applicability of the findings. This rationale has now been further clarified in the manuscript.*

**4) The lack of detailed information regarding postoperative complications**

**Answer:** *We refer to our response to reviewer (comment 3)*

**6) The ambiguous definition of “potentially curable recurrence.”
Answer:** *We refer to our response to reviewer (comment 1*)

**7) The authors should provide separate analyses for stage I-III and stage IV disease. If feasible, analyses by individual stage should also be presented. These analyses should include recurrence rates, potentially curable recurrence, non-curable recurrence, and the number needed to follow up (NNFU).**

**Answer:** *Thank you for this comment. We would like to clarify that analyses were already performed separately for stages I, II, III, and IV and were presented in the Supplementary Materials. These stage-specific analyses include recurrence rates, potentially curable recurrence, non-curable recurrence, and in Table 4 the corresponding NNFU estimates for each individual stage.*

*In response to the reviewer's suggestion, we additionally performed a Cox regression analysis stratified by tumour stage. Stratified analyses were performed to allow baseline hazards and covariate effects to vary across tumour stage subgroups. However, we were particularly interested in estimating the effect of tumour stage itself when included as a covariate in the multivariable model. Moreover, the unstratified multivariable model showed better predictive discrimination than the stratified model, with a Harrell’s C-index of 0.74 versus 0.62, respectively. Therefore, we selected the unstratified multivariable model as the definitive and most parsimonious model, as it both provided better predictive performance and allowed direct estimation of the effect of tumour stage*

**9) The handling of postoperative complications should be described in greater detail using the Clavien–Dindo classification. At minimum, the authors should consider separating minor and major complications in the analysis.**

**Answer:** *We refer to our response to reviewer (comment 3)*

**10) In addition, the competing-risk analysis should include cumulative incidence function estimates for all relevant competing events, including potentially curable recurrence, non-curable recurrence, and non-CRC death.**

**Answer:** *Thank you for this suggestion. Our study was specifically designed to assess the occurrence and detection of potentially curable recurrence and the corresponding efficiency of follow-up strategies. Although non-curable recurrence and non-CRC death are competing events, separate CIF estimates for these outcomes do not contribute to answering the primary clinical question and did not alter the interpretation of the primary endpoint. We therefore restricted our competing-risk analyses and reporting to potentially curable recurrence, which was the prespecified outcome of interest.*

**11) Finally, Table 3 should be thoroughly checked and corrected, including the hazard ratios, confidence intervals, p-values, variable coding, and reference categories.
Answer:** *We thank the reviewer for this comment. We carefully reviewed Table 3 and corrected the identified errors. Hazard ratios, confidence intervals, p-values, variable coding, and reference categories were rechecked against the original analyses and corrected where necessary. In response to the reviewer's suggestions, the presentation of the table has also been modified to improve clarity and consistency.*

**Review Comments to the Author**

**1. The abstract directly includes stage I-IV patients in the analysis, while the introduction and discussion primarily reference follow-up guidelines targeting stage I-III, creating inconsistency. It is recommended to**

a) clearly justify the inclusion of stage IV patients and

b) explain the rationale behind defining "curative intent

**Answer; clearly justify the inclusion of stage IV patients**.

*Thank you for this important comment. We agree that most published follow-up recommendations have traditionally focused on patients with stage I–III colorectal cancer. However, we included stage IV patients who underwent treatment of synchronous metastatic disease with curative intent because these patients are routinely enrolled in postoperative surveillance programmes in daily clinical practice after achieving a curative-intent status.*

*The rationale for surveillance in this population is comparable to that in stage I–III disease, namely the early detection of recurrent disease that may still be amenable to curative-intent treatment. Excluding these patients would therefore not reflect real-world clinical practice and would limit the applicability of our findings.*

*During the study period, specific recommendations for surveillance after curative treatment of synchronous metastatic disease were not yet explicitly incorporated in the Dutch colorectal cancer guideline. Nevertheless, these patients were routinely followed according to local clinical practice. Formal recommendations for this patient group have subsequently been incorporated into the Dutch guideline (March 2026), reflecting the growing recognition of structured surveillance in this population. To address the reviewer's concern, we have clarified the rationale for inclusion of stage IV patients in the Abstract, Introduction, Methods and Discussion sections*

***Answer:* explain the rationale behind defining "curative intent”.**

*Thank you for this comment. To improve clarity, we have revised the definition of potentially curable recurrence in the Methods section. The original definition was based on the treatment received and may have been interpreted ambiguously. We now explicitly define potentially curable recurrence as recurrent disease for which the multidisciplinary tumour board considered curative-intent treatment feasible.*

*The revised text now reads:*

*“The potentially curable recurrence group included patients who developed one or more recurrences for which the multidisciplinary tumour board considered curative-intent treatment feasible, including surgical resection, stereotactic ablative radiotherapy (SABR), or radiofrequency ablation (RFA).”*

**2. The article mainly cites literature from 2014–2018, lacking recent advances over the past 2–3 years on ctDNA and risk-adaptive follow-up, making it somewhat outdated.**

**Answer:** *We respectfully disagree that the manuscript is primarily based on outdated literature. Key references such as FACS and COLOFOL were deliberately included because they constitute the scientific foundation of current follow-up guidelines. Nevertheless, to address the reviewer’s concern and better reflect recent developments, we have added recently published data from the PRODIGE 13 trial to the Introduction and incorporated a recent ctDNA study in the Discussion. While these additions ensure that the manuscript reflects the current literature, they do not introduce new information that changes the interpretation of our findings.*

**3. The term "postoperative complications" is not differentiated by Clavien-Dindo grade, yet the subsequent discussion attempts to interpret their association with "curable recurrence," resulting in weak causal logic.**

**Answer:** *We thank the reviewer for this important observation. We agree that postoperative complications should not be considered a homogeneous entity. Therefore, complications have now been stratified according to the Clavien-Dindo classification and analysed as minor (Clavien-Dindo grade I-II) and major (Clavien-Dindo grade III-V) complications. The Results and Discussion sections have been revised* *accordingly****.***

**4. In the phrase "non-curable recurrence or death as competing events," the use of "or" is typically inappropriate in competing risks models—non-curable recurrence and death are not mutually exclusive. Please clarify whether these two events have been combined into a single composite competing event.**

**Answer:** *We thank the reviewer for this comment. The description was indeed unclear. Non-curable recurrence and non-CRC death were not combined into a single composite competing event. Rather, they were treated as separate competing events in the competing risk analysis. We have revised the Methods section in the abstract to clarify this and to avoid the suggestion that these outcomes were analysed as a combined endpoint.*

5. The article does not provide cumulative incidence function (CIF) curves or cumulative incidence rates for each competing event (non-curable recurrence, non-CRC death) in the competing risks model, thus preventing an assessment of the relative weight of "curable recurrence" within the overall risk profile.

**Answer:** *We refer to our response to editor (comment 10)*
