# Supplementary material for: Detection of potentially curable recurrence after colorectal cancer surgery in a real-world population
Source: PLoS One. 2026 Oct 30;21(10):e0361933. doi: 10.1371/journal.pone.0361933 (PMC13722166; doi:10.1371/journal.pone.0361933)
Supplement: Peer review history

# Peer review: Decision Letter 0

**DOI:** 10.1371/journal.pone.0361933.r001

**Authors:** Tsutomu Kumamoto

17 Jun 2026

Dear Dr. Schoenaker,

plosone@plos.org. When you're ready to submit your revision, log on to https://www.editorialmanager.com/pone/ and select the 'Submissions Needing Revision' folder to locate your manuscript file.

- A letter that responds to each point raised by the academic editor and reviewer(s). You should upload this letter as a separate file labeled 'Response to Reviewers'.
- A marked-up copy of your manuscript that highlights changes made to the original version. You should upload this as a separate file labeled 'Revised Manuscript with Track Changes'.
- An unmarked version of your revised paper without tracked changes. You should upload this as a separate file labeled 'Manuscript'.

If you would like to make changes to your financial disclosure, please include your updated statement in your cover letter. Guidelines for resubmitting your figure files are available below the reviewer comments at the end of this letter.

If applicable, we recommend that you deposit your laboratory protocols in protocols.io to enhance the reproducibility of your results. Protocols.io assigns your protocol its own identifier (DOI) so that it can be cited independently in the future. For instructions see: https://journals.plos.org/plosone/s/submission-guidelines#loc-laboratory-protocols. Additionally, PLOS ONE offers an option for publishing peer-reviewed Lab Protocol articles, which describe protocols hosted on protocols.io. Read more information on sharing protocols at https://plos.org/protocols?utm_medium=editorial-email&utm_source=authorletters&utm_campaign=protocols.

As the corresponding author, your ORCID iD is verified in the submission system and will appear in the published article. PLOS supports the use of ORCID, and we encourage all coauthors to register for an ORCID iD and use it as well. Please encourage your coauthors to verify their ORCID iD within the submission system before final acceptance, as unverified ORCID iDs will not appear in the published article. *Only*  the individual author can complete the verification step; PLOS staff *cannot*  verify ORCID iDs on behalf of authors.

We look forward to receiving your revised manuscript.

Kind regards,

Tsutomu Kumamoto

Academic Editor

PLOS One

Journal Requirements:

When submitting your revision, we need you to address these additional requirements.

1. Please ensure that your manuscript meets PLOS ONE's style requirements, including those for file naming. The PLOS ONE style templates can be found at https://journals.plos.org/plosone/s/file?id=wjVg/PLOSOne_formatting_sample_main_body.pdf and https://journals.plos.org/plosone/s/file?id=ba62/PLOSOne_formatting_sample_title_authors_affiliations.pdf

2. We note that you have indicated that there are restrictions to data sharing for this study. For studies involving human research participant data or other sensitive data, we encourage authors to share de-identified or anonymized data. However, when data cannot be publicly shared for ethical reasons, we allow authors to make their data sets available upon request. For information on unacceptable data access restrictions, please see http://journals.plos.org/plosone/s/data-availability#loc-unacceptable-data-access-restrictions.

Before we proceed with your manuscript, please address the following prompts:

a) If there are ethical or legal restrictions on sharing a de-identified data set, please explain them in detail (e.g., data contain potentially identifying or sensitive patient information, data are owned by a third-party organization, etc.) and who has imposed them (e.g., a Research Ethics Committee or Institutional Review Board, etc.). Please also provide contact information for a data access committee, ethics committee, or other institutional body to which data requests may be sent.

b) If there are no restrictions, please upload the minimal anonymized data set necessary to replicate your study findings to a stable, public repository and provide us with the relevant URLs, DOIs, or accession numbers. Please see http://www.bmj.com/content/340/bmj.c181.long for guidelines on how to de-identify and prepare clinical data for publication. For a list of recommended repositories, please see https://journals.plos.org/plosone/s/recommended-repositories. You also have the option of uploading the data as Supporting Information files, but we would recommend depositing data directly to a data repository if possible.

Please update your Data Availability statement in the submission form accordingly.

If the reviewer comments include a recommendation to cite specific previously published works, please review and evaluate these publications to determine whether they are relevant and should be cited. There is no requirement to cite these works unless the editor has indicated otherwise.

Additional Editor Comments:

The central clinical question of this manuscript—namely, how often postoperative surveillance can detect recurrences amenable to curative-intent treatment—is clinically meaningful. However, as also noted by the reviewer, several important issues currently limit the interpretation of the findings, including the inclusion of stage IV patients, the lack of detailed information regarding postoperative complications, and the ambiguous definition of “potentially curable recurrence.”

Therefore, I request that the authors substantially revise the analysis and presentation of the results. Although the overall cohort data may be presented, the authors should provide, at minimum, separate analyses for stage I–III and stage IV disease. If feasible, analyses by individual stage should also be presented. These analyses should include recurrence rates, potentially curable recurrence, non-curable recurrence, and the number needed to follow up (NNFU).

The handling of postoperative complications should be described in greater detail using the Clavien–Dindo classification. At minimum, the authors should consider separating minor and major complications in the analysis. In addition, the competing-risk analysis should include cumulative incidence function estimates for all relevant competing events, including potentially curable recurrence, non-curable recurrence, and non-CRC death.

Finally, Table 3 should be thoroughly checked and corrected, including the hazard ratios, confidence intervals, p-values, variable coding, and reference categories.

[Note: HTML markup is below. Please do not edit.]

Reviewers' comments:

Reviewer's Responses to Questions

**Comments to the Author**

1. Is the manuscript technically sound, and do the data support the conclusions?

Reviewer #1: Yes

**********

2. Has the statistical analysis been performed appropriately and rigorously?

Reviewer #1: Yes

**********

3. Have the authors made all data underlying the findings in their manuscript fully available?

The PLOS Data policy

Reviewer #1: Yes

**********

4. Is the manuscript presented in an intelligible fashion and written in standard English?

Reviewer #1: Yes

**********

Reviewer #1:

1. The abstract directly includes stage I-IV patients in the analysis, while the introduction and discussion primarily reference follow-up guidelines targeting stage I-III, creating inconsistency. It is recommended to clearly justify the inclusion of stage IV patients and explain the rationale behind defining "curative intent."

2. The article mainly cites literature from 2014–2018, lacking recent advances over the past 2–3 years on ctDNA and risk-adaptive follow-up, making it somewhat outdated.

3. The term "postoperative complications" is not differentiated by Clavien-Dindo grade, yet the subsequent discussion attempts to interpret their association with "curable recurrence," resulting in weak causal logic.

4. In the phrase "non-curable recurrence or death as competing events," the use of "or" is typically inappropriate in competing risks models—non-curable recurrence and death are not mutually exclusive. Please clarify whether these two events have been combined into a single composite competing event.

5. The article does not provide cumulative incidence function (CIF) curves or cumulative incidence rates for each competing event (non-curable recurrence, non-CRC death) in the competing risks model, thus preventing an assessment of the relative weight of "curable recurrence" within the overall risk profile.

**********

what does this mean?). If published, this will include your full peer review and any attached files.

If you choose “no”, your identity will remain anonymous but your review may still be made public.

**Do you want your identity to be public for this peer review?**  For information about this choice, including consent withdrawal, please see our Privacy Policy

Reviewer #1: No

**********

[NOTE: If reviewer comments were submitted as an attachment file, they will be attached to this email and accessible via the submission site. Please log into your account, locate the manuscript record, and check for the action link "View Attachments". If this link does not appear, there are no attachment files.]

To ensure your figures meet our technical requirements, please review our figure guidelines: https://journals.plos.org/plosone/s/figures

You may also use PLOS’s free figure tool, NAAS, to help you prepare publication quality figures: https://journals.plos.org/plosone/s/figures#loc-tools-for-figure-preparation.

NAAS will assess whether your figures meet our technical requirements by comparing each figure against our figure specifications.

# Author comment: Author response to Decision Letter 1

**DOI:** 10.1371/journal.pone.0361933.r002

13 Aug 2026

We thank the Editor and Reviewers for their careful evaluation of our manuscript and for their constructive comments. We have addressed all comments and suggestions raised by both the Editor and the Reviewers in the attached rebuttal letter. Corresponding revisions have been made throughout the manuscript, and all changes are detailed point-by-point in the rebuttal document.

Attachment Submitted filename: Rebuttal letter.docx

# Editor report: Decision Letter 1

**DOI:** 10.1371/journal.pone.0361933.r003

**Authors:** Tsutomu Kumamoto

21 Sep 2026

Dear Dr. Schoenaker,

Thank you for submitting your manuscript to PLOS One. After careful consideration, we feel that it has merit but does not fully meet PLOS One’s publication criteria as it currently stands. Therefore, we invite you to submit a revised version of the manuscript that addresses the points raised during the review process.

Please submit your revised manuscript by Nov 05 2026 11:59PM. If you will need more time than this to complete your revisions, please reply to this message or contact the journal office at plosone@plos.org. When you're ready to submit your revision, log on to https://www.editorialmanager.com/pone/ and select the 'Submissions Needing Revision' folder to locate your manuscript file.

- A letter that responds to each point raised by the academic editor and reviewer(s). You should upload this letter as a separate file labeled 'Response to Reviewers'.
- A marked-up copy of your manuscript that highlights changes made to the original version. You should upload this as a separate file labeled 'Revised Manuscript with Track Changes'.
- An unmarked version of your revised paper without tracked changes. You should upload this as a separate file labeled 'Manuscript'.

If you would like to make changes to your financial disclosure, please include your updated statement in your cover letter. Guidelines for resubmitting your figure files are available below the reviewer comments at the end of this letter.

If applicable, we recommend that you deposit your laboratory protocols in protocols.io to enhance the reproducibility of your results. Protocols.io assigns your protocol its own identifier (DOI) so that it can be cited independently in the future. For instructions see: https://journals.plos.org/plosone/s/submission-guidelines#loc-laboratory-protocols. Additionally, PLOS One offers an option for publishing peer-reviewed Lab Protocol articles, which describe protocols hosted on protocols.io. Read more information on sharing protocols at https://plos.org/protocols?utm_medium=editorial-email&utm_source=authorletters&utm_campaign=protocols.

As the corresponding author, your ORCID iD is verified in the submission system and will appear in the published article. PLOS supports the use of ORCID, and we encourage all coauthors to register for an ORCID iD and use it as well. Please encourage your coauthors to verify their ORCID iD within the submission system before final acceptance, as unverified ORCID iDs will not appear in the published article. *Only*  the individual author can complete the verification step; PLOS staff *cannot*  verify ORCID iDs on behalf of authors.

We look forward to receiving your revised manuscript.

Kind regards,

Tsutomu Kumamoto

Academic Editor

PLOS One

Journal Requirements:

If the reviewer comments include a recommendation to cite specific previously published works, please review and evaluate these publications to determine whether they are relevant and should be cited. There is no requirement to cite these works unless the editor has indicated otherwise.

Please review your reference list to ensure that it is complete and correct. If you have cited papers that have been retracted, please include the rationale for doing so in the manuscript text, or remove these references and replace them with relevant current references. Any changes to the reference list should be mentioned in the rebuttal letter that accompanies your revised manuscript. If you need to cite a retracted article, indicate the article’s retracted status in the References list and also include a citation and full reference for the retraction notice.

Additional Editor Comments:

Thank you for your careful revision of the manuscript. The manuscript has been substantially improved, and most of my previous concerns have been adequately addressed. I have only two remaining comments before the manuscript can be considered for acceptance.

1. Cumulative incidence functions for the competing events

This comment relates to Reviewer 1, Comment 10, and Reviewer 2, Comment 5.

The Methods state that the cumulative incidence function (CIF) was used to quantify the marginal probability of each competing event over time. However, the Results report the CIF only for potentially curable recurrence.

In the response letter, the authors state that separate CIF estimates for non-curable recurrence and non-CRC death do not contribute to the primary clinical question. However, because the Methods explicitly state that the CIF was used for each competing event, the corresponding results should also be reported. Whether these competing-event estimates contribute to the clinical interpretation should be assessed based on the observed results rather than assumed a priori.

Therefore, please provide the CIF estimates for non-curable recurrence and non-CRC death in the Results.

2. Interpretation of “direct therapeutic benefit”

The Conclusion states that 8.3% of patients “derive direct therapeutic benefit from standard follow-up.” However, the 8.3% represents the cumulative incidence of potentially curable recurrence and does not directly demonstrate a therapeutic benefit attributable to surveillance itself. Please revise this wording so that the conclusion more precisely reflects the outcome actually measured in the study.

[NOTE: If reviewer comments were submitted as an attachment file, they will be attached to this email and accessible via the submission site. Please log into your account, locate the manuscript record, and check for the action link "View Attachments". If this link does not appear, there are no attachment files.]

To ensure your figures meet our technical requirements, please review our figure guidelines: https://journals.plos.org/plosone/s/figures

You may also use PLOS’s free figure tool, NAAS, to help you prepare publication quality figures: https://journals.plos.org/plosone/s/figures#loc-tools-for-figure-preparation.

NAAS will assess whether your figures meet our technical requirements by comparing each figure against our figure specifications.

# Author comment: Author response to Decision Letter 2

**DOI:** 10.1371/journal.pone.0361933.r004

15 Oct 2026

We would like to sincerely thank you for your careful second evaluation of our manuscript and for your constructive feedback. We are pleased that only a few minor issues remained to be addressed. We have carefully considered both comments and revised the manuscript accordingly. Detailed responses to each comment are provided in the rebuttal lettre..

Attachment Submitted filename: Rebuttal letter 2.docx

# Editor report: Decision Letter 2

**DOI:** 10.1371/journal.pone.0361933.r005

**Authors:** Tsutomu Kumamoto

19 Oct 2026

Detection of potentially curable recurrence after colorectal cancer surgery in a real-world population

PONE-D-26-09874R2

Dear Dr. Ivonne J.H. Schoenaker,

We’re pleased to inform you that your manuscript has been judged scientifically suitable for publication and will be formally accepted for publication once it meets all outstanding technical requirements.

Within one week, you’ll receive an e-mail detailing the required amendments. When these have been addressed, you’ll receive a formal acceptance letter and your manuscript will be scheduled for publication.

An invoice will be generated when your article is formally accepted. Please note, if your institution has a publishing partnership with PLOS and your article meets the relevant criteria, all or part of your publication costs will be covered. Please make sure your user information is up-to-date by logging into Editorial Manager at Editorial Manager® and clicking the ‘Update My Information' link at the top of the page. For questions related to billing, please contact billing support.

If your institution or institutions have a press office, please notify them about your upcoming paper to help maximize its impact. If they’ll be preparing press materials, please inform our press team as soon as possible -- no later than 48 hours after receiving the formal acceptance. Your manuscript will remain under strict press embargo until 2 pm Eastern Time on the date of publication. For more information, please contact onepress@plos.org.

Kind regards,

Tsutomu Kumamoto

Academic Editor

PLOS One

Additional Editor Comments (optional):

Reviewers' comments:

# Editor report: Acceptance letter

**DOI:** 10.1371/journal.pone.0361933.r006

**Authors:** Tsutomu Kumamoto

PONE-D-26-09874R2

PLOS One

Dear Dr. Schoenaker,

I'm pleased to inform you that your manuscript has been deemed suitable for publication in PLOS One. Congratulations! Your manuscript is now being handed over to our production team.

At this stage, our production department will prepare your paper for publication. This includes ensuring the following:

* All references, tables, and figures are properly cited

* All relevant supporting information is included in the manuscript submission,

* There are no issues that prevent the paper from being properly typeset

You will receive further instructions from the production team, including instructions on how to review your proof when it is ready. Please keep in mind that we are working through a large volume of accepted articles, so please give us a few days to review your paper and let you know the next and final steps.

Lastly, if your institution or institutions have a press office, please let them know about your upcoming paper now to help maximize its impact. If they'll be preparing press materials, please inform our press team within the next 48 hours. Your manuscript will remain under strict press embargo until 2 pm Eastern Time on the date of publication. For more information, please contact onepress@plos.org.

You will receive an invoice from PLOS for your publication fee after your manuscript has reached the completed accept phase. If you receive an email requesting payment before acceptance or for any other service, this may be a phishing scheme. Learn how to identify phishing emails and protect your accounts at https://explore.plos.org/phishing.

If we can help with anything else, please email us at customercare@plos.org.

Thank you for submitting your work to PLOS One and supporting open access.

Kind regards,

PLOS One Editorial Office Staff

on behalf of

M.D., Ph.D. Tsutomu Kumamoto

Academic Editor

PLOS One
